# Sex Difference in Brain Responses During Short Abstinence in People With Internet Gaming Disorder

**DOI:** 10.1111/adb.70145

**Published:** 2026-03-25

**Authors:** Shaoyu Cui, Xuefeng Xu, Guangheng Dong

**Affiliations:** ^1^ Department of Psychology Yunnan Normal University Kunming Yunnan China; ^2^ Faculty of Education Yunnan Normal University Kunming Yunnan China

**Keywords:** functional connectivity, internet gaming disorder, sex difference, short abstinence

## Abstract

Withdrawal or the adverse response to abstinence is a significant marker of addiction; however, the neural features of internet gaming disorder (IGD), especially the effects of sex under abstinence, have rarely been examined. This study aimed to examine brain reactions in IGD patients after short‐term abstinence and the differences between the sexes. Thirty males and 30 females with IGDs and 30 males and 30 females recreational game users (RGUs) were recruited. Resting‐state fMRI data were collected after 1.5 h without gaming. In the IGD and RGU groups, we found atypical brain areas with concurrent degree centrality (DC) and regional homogeneity (ReHo) changes. We then performed functional connectivity (FC) analysis and two‐factor ANOVA on these regions to compare IGD and RGU and test for sex differences. Compared with RGUs, IGD subjects presented abnormal cerebral areas with concurrent DC and ReHo abnormalities. After short‐term abstinence, IGD and RGU patients presented abnormal prefrontal lobe and insula FC values. Subsequent sex difference analyses focused on the superior frontal gyrus (SFG), middle frontal gyrus (MFG), inferior frontal gyrus (IFG) and insula. ANOVA followed by FDR‐corrected post hoc comparisons revealed that IGD males exhibited significantly greater prefrontal and insula FC than females after short‐term abstinence. Specifically, males showed markedly enhanced FC in multiple prefrontal regions and the insula, with effect sizes (Cohen's *d*) ranging from medium to large, confirming both the efficacy and reliability of the observed differences. Compared with RGUs, IGD patients presented FC changes in executive control and reward processing brain regions. With respect to sex differences, short‐term abstinence may have altered cognitive control functions more in males than in females and increased internet gaming severity in males. These findings suggest that males are more susceptible to IGD.

## Graphical abstract

Short‐term abstinence in internet gaming disorder (IGD) reveals altered functional connectivity in executive control and reward brain regions compared to recreational users. Males with IGD show stronger functional connectivity between frontal and insular regions than females, indicating greater disruption in cognitive control. These findings suggest that males may be more vulnerable to IGD during short‐term abstinence.

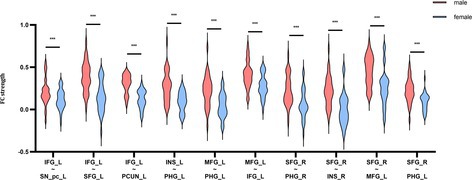

## 1 Introduction

Online gaming is a preferred form of amusement among contemporary youth. Nevertheless, uncontrolled gaming behaviours adversely affect physical and mental well‐being, ultimately resulting in internet gaming disorder (IGD). As a putative type of behavioural addiction, IGD is characterized as excessive, addicted or pathological online gaming [1]. The prevalence of IGD is 8.8% in adolescents and 10.4% in young adults [2]. Despite the ongoing debate surrounding the definition of IGD, the elements that lead to addiction and treatment approaches, it is unequivocal that IGD can profoundly affect an individual's physical and emotional well‐being [3, 4, 5, 6]. Research indicates that individuals with IGD demonstrate heightened depressive symptoms, anxiety, tension, diminished life satisfaction, impaired concentration and reduced sleep quality relative to their counterparts [7, 8].

Neuroimaging studies have identified significant alterations in functional brain networks associated with IGD. Functional connectivity (FC) values between brain areas associated with executive control activities and those linked to reward processing functions were also identified as aberrant. For example, one study reported that reduced FC was observed between the bilateral nucleus accumbens and the left dorsolateral prefrontal cortex (DLPFC) in individuals with IGD [9]. Another study reported that the IGD group displayed decreased FC between the OFC and dorsal striatum compared with control subjects [10]. A reduced FC strength of the DLPFC‐caudate and OFC‐nucleus accumbens circuits was observed between the IGD group and the control group [11]. Additionally, a previous study revealed that the IGD group had relatively decreased dynamic FC between the right DLPFC and the left insula, right putamen and left precentral gyrus [12]. IGD subjects had greater resting‐state FC between the orbital frontal cortex and inferior frontal gyrus than did controls [13].

Investigating sex‐related variations and similarities in IGD is essential for formulating effective policies, preventative strategies and treatment initiatives [14]. Research indicates that males exhibit heightened sensitivity to gaming‐related rewards and stronger gaming cravings during acute abstinence than do females [15]. In terms of cerebral characteristics, IGD males presented greater striatal activation than IGD females [16, 17]; the lenticular nucleus in IGD males presented superior FC compared with that in IGD females [18]. According to anatomical physiology, the dorsal striatum encompasses the caudate nucleus and the putamen, which are further divided into the putamen and pallidum. During gameplay, a reduction in FC between the DLPFC and the superior frontal gyrus (SFG), alongside an increase in FC between the dorsal striatum and the thalamus, was observed exclusively in IGD males, with this phenomenon absent in IGD females [19]. Although IGD does not involve substance intake, it has been proposed that IGD and substance use disorders may share commonalities, especially with respect to neuronal pathways in the brain [20, 21, 22]. Compared with females in alcohol use disorders, males presented greater FC activation in the medial prefrontal cortex, anterior cingulate cortex and amygdala [23]. In studies with cocaine use disorders, females displayed greater FC activation of the prefrontal cortex than did males [24]. In individuals with tobacco use disorders, elevated FC activation was observed in the SFG and posterior cingulate cortex in females [25].

Withdrawal symptoms, which emerge during abstinence, originate from substance addictions. They are defined as ‘the unpleasant feeling states and/or physical effects that occur when a specific activity ceases or is suddenly reduced’ [26, 27]. It is traditionally deemed absent in behavioural addictions [28]; however, subsequent research has identified a dysregulated dopamine system as prevalent in both behavioural and substance addictions [29], suggesting that abstinence may serve as a crucial diagnostic criterion in behavioural addictions. Prior investigations predominantly focused on IGD abstinence through interviews, questionnaires, self‐reports, psychometric assessments or case studies, with fewer studies addressing the neurological mechanisms of the brain in IGD abstinence [30]. Dong [19] employed fMRI scans to assess alterations in FC among the DLPFC, striatum and SFG during enforced abstinence; [31] noted changes in FC between the striatum and thalamus during abstinence. Concurrently, FC between the dorsal striatum and the posterior cingulate gyrus, as well as between the medial prefrontal cortex and the hippocampus, is markedly diminished in a study with tobacco use disorders after abstinence [32]. In individuals with alcohol addiction, FC within the left prefrontal–striatal network significantly decreased following alcohol abstinence [33]. However, few studies investigated the distinctions in IGD after short‐term abstinence and the ensuing sex disparities.

Regional homogeneity (ReHo) denotes the extent of similarity between the time series of a particular voxel and its adjacent voxels [34]. It functions as a resting‐state fMRI metric and is frequently employed to identify the temporal synchronization of BOLD signals in particular brain areas [35]. Unlike conventional techniques, ReHo optimizes the characteristics of the resting state by selecting areas of interest [36, 37]. Degree centrality (DC), a graph‐theoretic metric of network structure, indicates the quantity of transient functional connections between a specific brain area and the overall connectivity matrix of the brain and is extensively employed to identify alterations in resting‐state functional networks [38]. It may thus evaluate the degree to which a node influences the entire brain and synthesizes input from functionally distinct brain regions [39]. The combination of ReHo and DC facilitates enhanced monitoring of synchronization between specific brain regions and others, improving the characterization of the intrinsic activity of the brain [40, 41].

Using delayed brain imaging scans, this study investigated short‐term abstinence effects by comparing FC patterns between IGD and RGU, while exploring sex‐related FC differences in IGD. We anticipate that disparities in the activity of brain areas linked to cognitive regulation and reward processing will be observed between IGD and RGU. Differences in the activity of brain areas linked to cognitive control and reward processing may also be observed between IGD males and IGD females.

## 2 Methods

### 2.1 Subjects

The Human Investigation Committee at Yunnan Normal University approved the study. Informed consent was acquired in writing from all participants. The complete experimental protocol adhered to the Declaration of Helsinki.

Sixty individuals with IGD (30 males and 30 females) and 60 individuals with RGU (30 males and 30 females) were recruited through advertisements. An a priori power analysis (one‐way ANOVA, *f* = 0.25, 80% power, *α* = 0.05) indicated a required sample size of 52; our final sample of 120 (60 per group) ensures adequate statistical power for the primary group comparisons. The RGU group engaged in online gaming but did not shift from nonaddiction to addiction [42, 43]. Compared with the general control group, the RGUs had greater gaming experience and extended gaming duration. All the subjects were evaluated via a structured mental interview [44], omitting other mental diseases, such as depression, anxiety, schizophrenia and substance use disorders. Moreover, participants were instructed to abstain from consuming drugs and substances (e.g., coffee, tea) on the day of the experiment.

The criteria for screening addiction were derived from the young online internet addiction test (IAT) and the nine diagnostic criteria for internet gaming disorder established by the DSM‐5 Committee [1, 45]. The criteria for inclusion in IGDs are as follows: (1) IAT score exceeding 50; (2) DSM‐5 score surpassing 5; (3) having played internet games for at least 1 year. RGU participants were required to engage in regular online gaming without developing addiction. Specifically, they matched IGD subjects in gaming frequency over the past year while demonstrating no physical or psychological dependence through clinical assessments (IAT scores under 50 and DSM‐5 scores meeting fewer than 5 of 9). Table 1 presents the demographic data of the two groups.

**TABLE 1:** Demographic information and group differences.

	IGD (*n* = 60)		RGU (*n* = 60)		Main effect (group)	Main effect (sex)	Interaction (Group × Sex)
	M (*n* = 30)	F (*n* = 30)	M (*n* = 30)	F (*n* = 30)	*F*, *P*, partial *η* ^*2*^	*F*, *P*, partial *η* ^*2*^	*F*, *P*, partial *η* ^*2*^
Age (mean ± SD)	21.37 ± 2.66	20.90 ± 2.187	21.10 ± 2.67	20.90 ± 2.02	*F* = 0.092, *p =* 0.762, *η* ^2^ < 0.001	*F* = 0.578, *p =* 0.449, *η* ^2^ = 0.005	*F* = 0.092, *p = 0*.762, *η* ^2^ < 0.001
IAT	66.87 ± 10.47	65.17 ± 7.88	38.10 ± 9.29	40.33 ± 11.87	*F* = 216.012, *p* < 0.001, *η* ^2^ = 0.651	*F* = 0.021, *p =* 0.884, *η* ^2^ < 0.001	*F* = 1.163, *p =* 0.283, *η* ^2^ = 0.01
DSM	6.23 ± 1.07	6.03 ± 1.16	2.17 ± 1.68	2.77 ± 1.50	*F* = 212.796, *p* < 0.001, *η* ^2^ = 0.647	*F* = 0.633, *p =* 0.428, *η* ^2^ = 0.005	*F* = 2.532, *p* = 0.114, *η* ^2^ = 0.021
GTPW	7.80 ± 3.16	9.37 ± 4.15	5.63 ± 2.58	5.17 ± 2.48	*F* = 30.43, *p* < 0.001, *η* ^2^ = 0.208	*F* = 0.908, *p =* 0.343, *η* ^2^ = 0.008	*F* = 3.104, *p =* 0.081, *η* ^2^ = 0.026
GH	3.83 ± 0.46	3.60 ± 0.81	3.60 ± 0.97	3.70 ± 0.60	*F* = 0.246, *p =* 0.621, *η* ^2^ = 0.002	*F* = 0.246, *p =* 0.621, *η* ^2^ = 0.002	*F* = 1.538, *p =* 0.217, *η* ^2^ = 0.013
Craving	53.93 ± 16.14	54.53 ± 16.86	33.97 ± 7.78	37.20 ± 20.26	*F* = 41.085, *p* < 0.001, *η* ^2^ = 0.262	*F* = 0.434, *p =* 0.511, *η* ^2^ = 0.004	*F* = 0.205, *p =* 0.652, *η* ^2^ = 0.002

Abbreviations: DSM, Diagnostic and Statistical Manual of Mental Disorders‐5; F, female; GH, gaming history (years); GTPW, gaming time per week (hours); IAT, internet addiction test; IGD, internet gaming disorder; M, male; RGU, recreational game user.

### 2.2 The Creation of Short Abstinence

Upon arrival at the laboratory, all participants were directed to complete the questionnaire and were advised to refrain from engaging in any online gaming activities. To facilitate the temporary cessation of gaming behaviours among IGD and RGU, each participant was mandated to remain in a quiet room devoid of any gaming devices (including all electronic devices) for approximately 90 min prior to the functional MRI scan. This duration of abstinence was chosen based on evidence from nicotine dependence research, which shows that craving—a core withdrawal symptom—can significantly increase after only 1 h of cigarette deprivation [46, 47]. Furthermore, our research group has previously employed a comparable 90‐min abstinence protocol in IGD studies to effectively induce craving states and examine related neural alterations [48].

### 2.3 fMRI Data Acquisition

Each participant was placed in a supine position, with the head securely immobilized using a belt and foam pads to reduce head movement. All the participants were instructed to close their eyes, remain motionless and refrain from concentrating on any specific thoughts during the scan. The fMRI resting data were acquired via a 3T MRI system (Siemens Trio). The specific parameters used were as follows: repetition time (TR) = 2000 ms, 33 interleaved slices, echo time (TE) = 30 ms, thickness = 3.0 mm, flip angle = 90°, field of view (FOV) = 220 mm × 220 mm and matrix = 64 × 64.

### 2.4 Data Preprocessing

The preprocessing of the rs‐fMRI data was completed in Statistical Parametric Mapping (SPM12, http://www.fil.ion.ucl.ac.uk/spm), and data processing and analysis were performed at Brain Imaging (DPABI, http://rfmri.org/dpabi) [49]. The preprocessing of the rs‐fMRI data included the following steps: (1) The quality of the raw rs‐fMRI data for all the participants was examined. (2) The raw data were transformed from the Digital Imaging and Communication in Medicine (DICOM) format to the Neuroimaging Informatics Technology Initiative (NIFTI) format. (3) The initial 10 volumes were discarded to maintain magnetic field stabilization. (4) Slice timing. (5) Realignment (excluding subjects with maximum head motion > 2.5 mm or rotation > 2.5°). (6) Regressing nuisance covariates, including the signal noise of white matter, cerebrospinal fluid and Friston‐24 head motion parameters [50]. (7) Filtering by a bandpass filter with a frequency range of 0.01–0.1 Hz before the calculation of ReHo and DC. (8) Smooth.

### 2.5 ROI Selection

**FIGURE 1:**
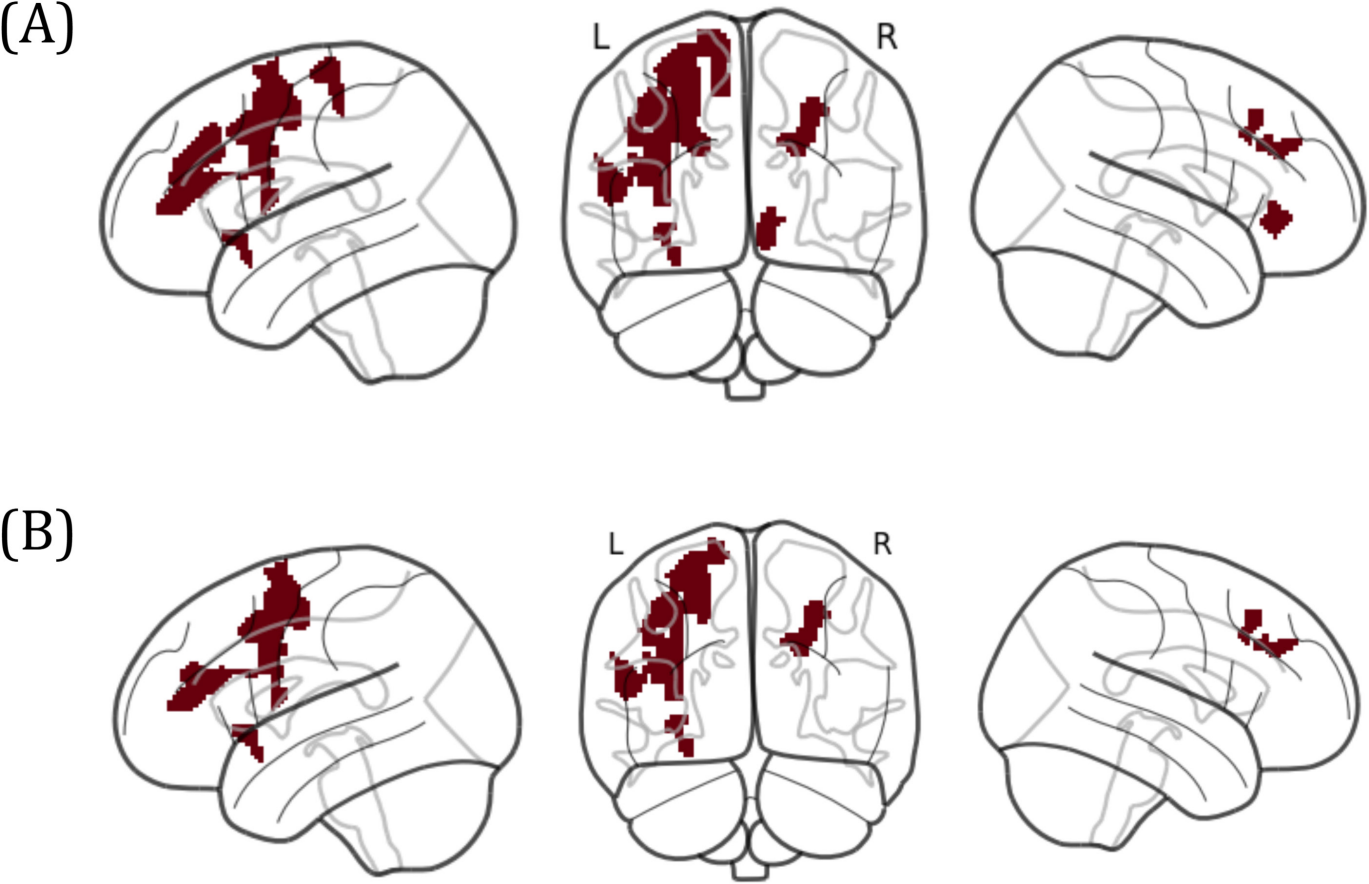
The ROI selections in the current data analyses. Schematic representation of the brain in the region of interest (ROI) for FC analysis based on degree centrality (DC) and regional homogeneity (ReHo) results. Panel (A) is the ROIs used to compare IGDs and RGUs, while Panel (B) is the ROIs used for exploring at sex differences in IGDs.

Initially, we calculated the ReHo and DC values for the IGD and RGU groups and subsequently conducted independent sample *t*‐test using the SPM12 toolbox. The DC and ReHo results were subsequently superimposed using DPABI to identify overlapping brain regions with a threshold of *p* < 0.05 and a minimum of 30 voxels. Afterward, we discovered that the results of IFG_L, MFG_L, SFG_L/R, OFC_R, nucleus accumbens_L, insula_L and paracentral_L were significant, as shown in Figure 1A. Therefore, we utilized the significant overlapping brain regions identified using DC and ReHo as regions of interest for FC analyses to examine the differences between the IGD and RGU groups. Based on their established roles in executive control and reward processing [51, 52] and their identification as abnormal in our prior analysis, we selected four of these brain regions: IFG_L, SFG_R, insula_L and MFG_L—as ROIs to maintain experimental continuity in the sex difference investigation, as illustrated in Figure 1B.

### 2.6 Statistical Analysis

For the FC analysis, an independent sample *t*‐test was used to compare the IGD and RGU groups. The significance threshold was established at *p* = 0.05, and multiple comparisons were adjusted using FDR correction. A 2 (sex: male, female) × 2 (group: IGD, RGU) ANOVA was subsequently conducted to investigate the interactions between sex and group in brain connectivity. Initially, we identified brain regions that exhibited substantial connections with the ROIs previously used, followed by the discovery of significant Group * Sex interactions in connectivity strengths across each condition. We subsequently extracted these values and performed simple‐effect tests on the areas exhibiting significant interactions to elucidate the nature of such interactions. The FC maps were generated via Prism 9.5 software (Prism 9.5.0 Release Notes). Then, we computed correlations between the retrieved FC values and self‐reported measures of internet gaming severity.

## 3 Results

### 3.1 Independent Sample *t*‐Test and ROI‐Based Analysis of the Interaction Effect

**FIGURE 2:**
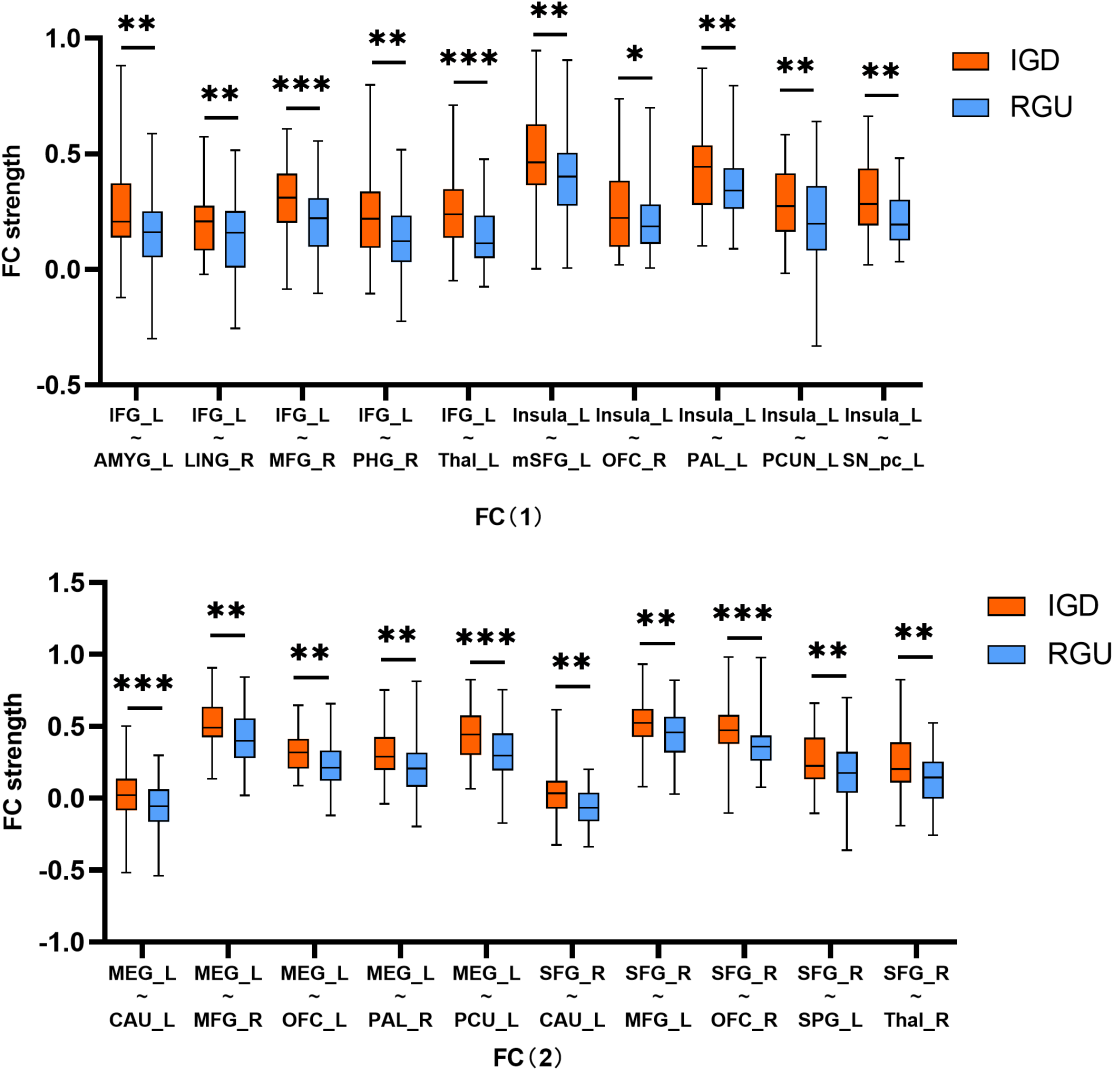
The results of independent sample *t*‐test between IGD and RGU. Both FC(1) and FC(2) are the function of the Internet game disorder and Recreational game use comparison results. AMYG: amygdala; CAU: caudate; IFG: inferior frontal gyrus; LING: lingual gyrus; MFG: middle frontal gyrus; mSFG: medial superior frontal gyrus; OFC: orbital gyrus; PAL: pallidum; PCUN: precuneus; PHG: parahippocampal gyrus; SN_pc: substantia nigra, pars compacta; SPG: superior parietal gyrus; Thal: thalamus. * representative *p* < 0.05, ** representative *p* < 0.01, *** representative *p* < 0.001.

The findings of an independent sample *t*‐test indicated that, after the short abstinence condition, in comparison with RGUs, individuals with IGD presented aberrant functional connections among the IFG, insula, MFG and SFG, and values were significant if *p* < 0.05. The specific values are presented in Figure 2.

### 3.2 Simple‐Effect Analysis of Sex Differences

Two‐factor ANOVA indicated significant FC involving IFG_L as the seed point, with notable interactions observed between IFG_L and MFG_L, PHG_R, SN_pc_L, MFG_R, SFG_L and Precuneus_L. A significant interaction in FC was observed between insula_L and SFG_R, PHG_L, utilizing insula_L as the seed point. A notable interaction was observed in the FC between MFG_L and PHG_L, IFG_L, with MFG_L serving as the seed point. Significant interactions in FC with SFG_R as the seed point were also observed between SFG_R and PHG_R, insula_R, precentral gyrus_R, MFG_L and ACC_L, as well as between SFG_R and PHG_L. More specific values are presented in Table S1.

**FIGURE 3:**
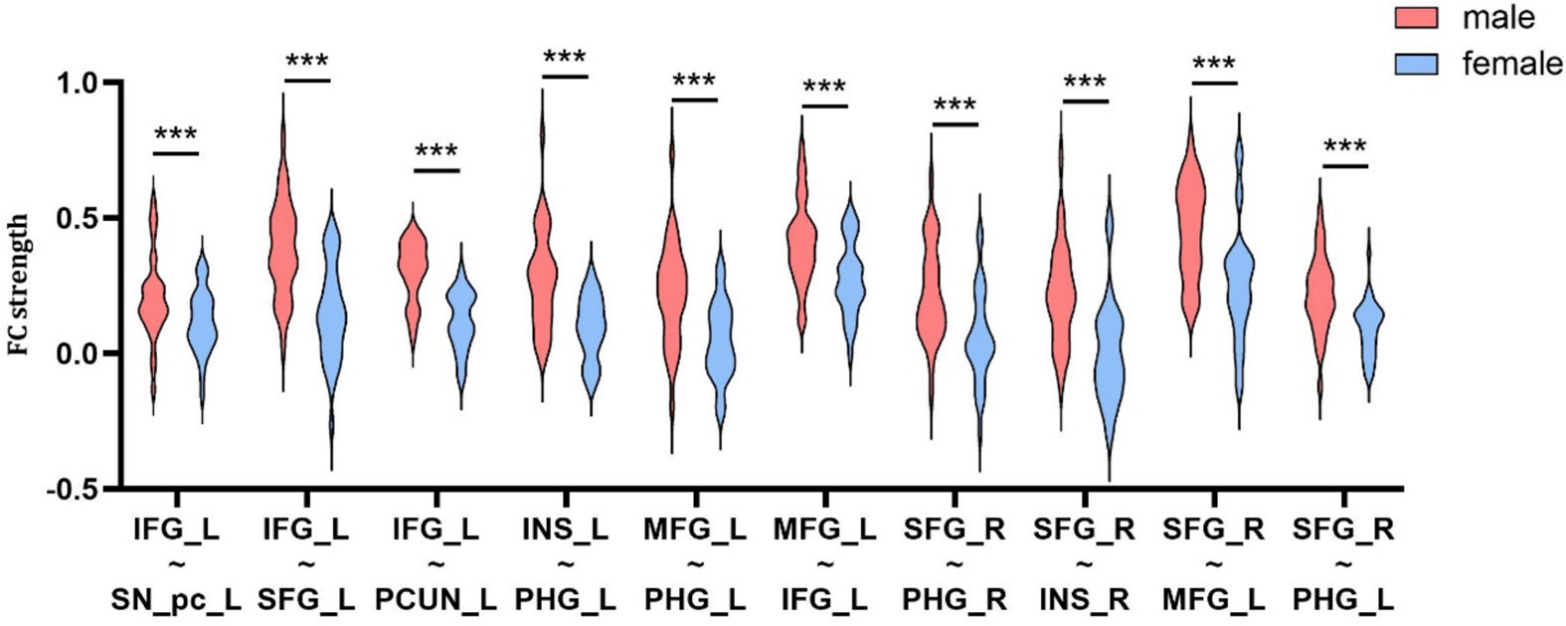
Results of simple effects analysis within the IGD group. The results of simple effects on sex differences within IGD groups. IFG: inferior frontal gyrus; INS: insula; MFG: middle frontal gyrus; PCUN: precuneus; PHG: parahippocampal gyrus; SFG: superior frontal gyrus; SN_pc: substantia nigra, pars compacta. *** representative *p* < 0.001.

Subsequent analyses of simple effects with FDR correction revealed that, in comparison with IGD females, IGD males presented markedly enhanced FC in the IFG_L (seed point) and MFG_L (*F*_1,116_ = 6.506; *p*_*fdr*_ = 0.015; Cohen's *d* = 0.658; 95% CI, −0.044 to 1.361), MFG_R (*F*_1,116_ = 12.406; *p*_*fdr*_ = 0.003; Cohen's *d* = 0.91; 95% CI, 0.198–1.621), SFG_L (*F*_1,116_ = 25.425; *p*_*fdr*_ = 0.000; Cohen's *d* = 1.302; 95% CI 0.572–2.032) and precuneus_L (*F*_1,116_ = 25.804; *p*_*fdr*_ = 0.000; Cohen's *d* = 1.311; 95% CI, 0.581–2.042), with males demonstrating significantly greater connectivity than females. Males exhibited much enhanced FC in Insula_L (seed point) and PHG_L (*F*_1,116_ = 20.777; *p*_*fdr*_ = 0.000; Cohen's *d* = 1.178; 95% CI, 0.454–1.901) as well as SFG_R (*F*_1,116_ = 11.881; *p*_*fdr*_ = 0.003; Cohen's *d* = 0.89; 95% CI, 0.18–1.601). Compared with females, males had markedly enhanced FC in MFG_L (seed point) and PHG_L (*F*_1,116_ = 17.156; *p*_*fdr*_ = 0.000; Cohen's *d* = 1.069; 95% CI, 0.351–1.787), as well as in IFG_L (*F*_1,116_ = 13.782; *p*_*fdr*_ = 0.000; Cohen's *d* = 0.958; 95% CI, 0.245–1.672). Moreover, males presented significantly enhanced FC than females between SFG_R (seed point) and PHG_R (*F*_1,116_ = 13.843; *p*_*fdr*_ = 0.000; Cohen's *d* = 0.961; 95% CI, 0.247–1.674), Insula_R (*F*_1,116_ = 19.125; *p*_*fdr*_ = 0.000; Cohen's *d* = 1.13; 95% CI, 0.409–1.851), precentral gyrus_R (*F*_1,116_ = 10.541; *p* = 0.003; Cohen's *d* = 0.838; 95% CI, 0.145–1.531), MFG_L (*F*_1,116_ = 13.185; *p*_*fdr*_ = 0.000; Cohen's *d* = 0.937; 95% CI, 0.225–1.65), ACC_L (*F*_1,116_ = 2.973; *p*_*fdr*_ = 0.087; Cohen's *d* = 0.445; 95% CI, −0.252 to 1.143) and PHG_L (*F*_1,116_ = 15.08; *p*_*fdr*_ = 0.000; Cohen's *d* = 1.003; 95% CI, 0.288–1.718). Specific values are presented in Figure 3 and Table S2. No sex disparities were observed in the RGU group. The distinctions between IGD and RGU for males, as well as for females, are presented in Table S3.

### 3.3 Correlation Analysis

**FIGURE 4:**
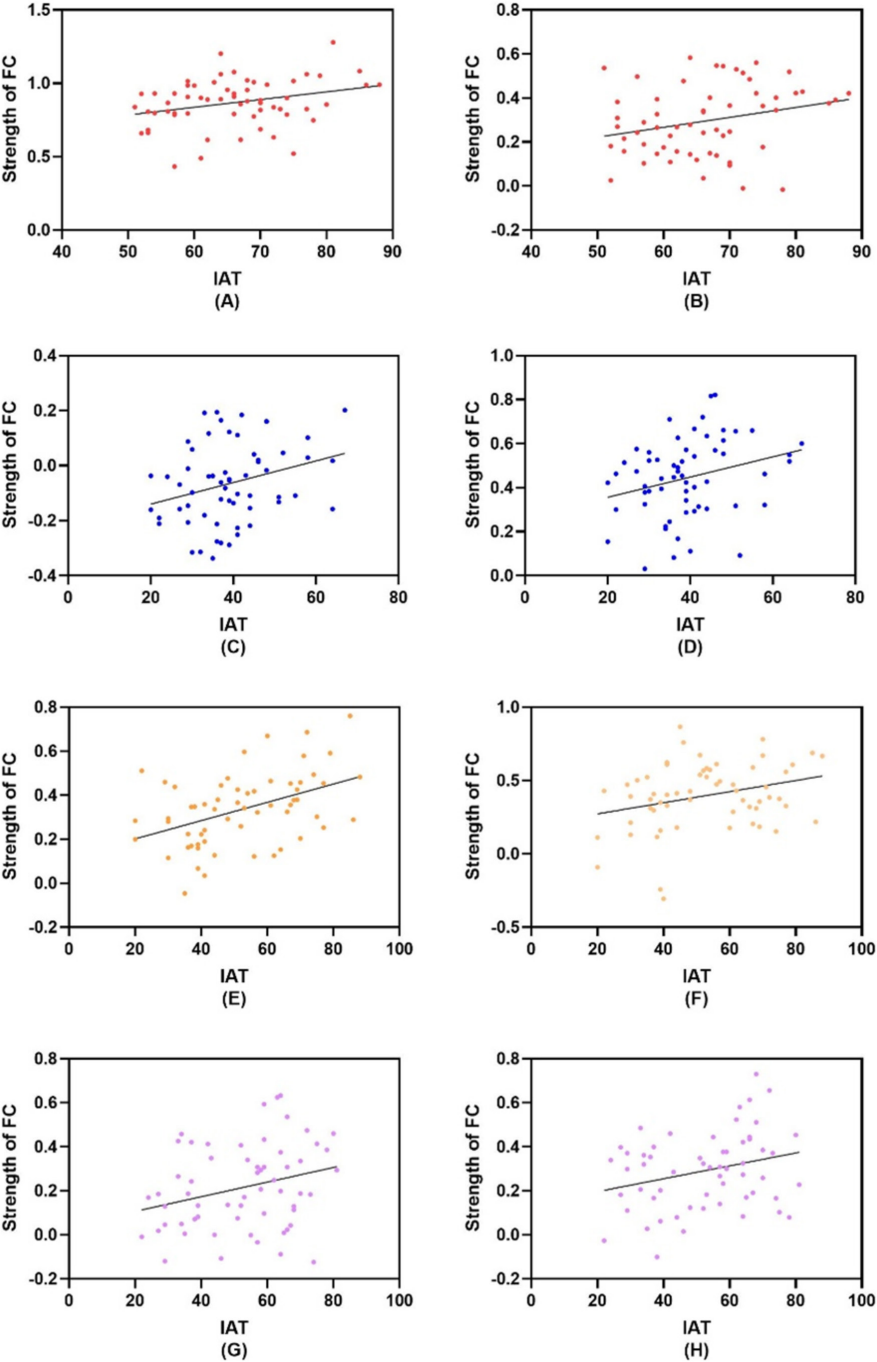
Correlation analysis between functional connectivity based on ROI and behavioural data. (A) Correlation result between IAT and FC strength between OFC_R and ACC_R in IGD group. (B) Correlation result between IAT and FC strength between insula_L and precuneus_L in IGD group. (C) Correlation result between IAT and FC strength between SFG_R and caudate_L in RGU group. (D) Correlation result between IAT and FC strength between SFG_R and MFG_L in RGU group. (E) Correlation result between IAT and FC strength between MFG_L and IFG_L in males. (F) Correlation result between IAT and FC strength between MFG_L and SFG_R in males. (G) Correlation result between IAT and FC strength between MFG_L and IFG_L in females. (H) Correlation result between IAT and FC strength between SFG_R and MFG_R. FC: functional connectivity; IAT, internet addiction test; IFG: inferior frontal gyrus; MFG: middle frontal gyrus; OFC: orbital gyrus; SFG: superior frontal gyrus.

A significant positive correlation (*r* = 0.298; *p* = 0.021; 95% CI, 0.048–0.513) between the IAT and FC strength between the OFC_R and ACC_R was detected in the IGD group. The same results were found for insula_L and precuneus_L (*r* = 0.264; *p* = 0.042; 95% CI, 0.01–0.485). A significant positive correlation (*r* = 0.289; *p* = 0.025; 95% CI, 0.038–0.506) between the IAT and FC strength was detected between the SFG_R and caudate_L in the RGU group. The same results were found for SFG_R and MFG_L (*r* = 0.273; *p* = 0.035; 95% CI, 0.021–0.493). We found a significant correlation between the craving score and FC strength between insula_L and MFG_R (*r* = −0.284; *p* = 0.028; 95% CI, −0.501 to −0.023), SFG_L and SFG_R (*r* = −0.286; *p* = 0.027; 95% CI, −0.503 to −0.034), OFC_R and OFC_L (*r* = −0.296; *p* = 0.021; 95% CI, −0.512 to −0.046) and OFC_R and putamen_R (*r* = −0.269; *p* = 0.038; 95% CI, −0.489 to −0.016) in the IGD group. We also found a significant positive correlation between the IAT and FC between MFG_L and IFG_L (*r* = 0.443; *p* = 0.000; 95% CI, 0.213–0.626) in males, as well as between SFG_R and MFG_L (*r* = 0.292; *p* = 0.023; 95% CI, 0.041–0.508). There was a significant positive correlation between the IAT score and FC strength between the IFG_L and MFG_L in females (*r* = 0.277; *p* = 0.032; 95% CI, 0.025–0.496). The same results were found for SFG_R and MFG_R (*r* = 0.275; *p* = 0.033; 95% CI, 0.023–0.495). Specific values are presented in Figure 4.

## 4 Discussion

This study employed resting‐state fMRI to compare IGD and RGU during short‐term abstinence, as well as male and female differences in the IGD and RGU groups. Compared with RGU, IGD demonstrated aberrant functional connections between executive control, reward processing, and associated brain areas. Compared with females, IGD males presented greater functional connections between executive control, reward processing, and executive control.

### 4.1 Enhanced Coupling in Brain Regions Responsible for Executive Control

This study revealed that, compared with RGU, IGD significantly increased FC values in the IFG_L. The IFG is a vital region for substance dependence and behavioural addiction [53], contributing to the development of subjective feelings during decision‐making [54, 55], and has also been associated with risky and adverse decision‐making [56, 57]. This enhancement may reflect a compensatory effort to regulate abstinence‐induced urges or, alternatively, a training effect from extensive gaming.

### 4.2 Enhanced Coupling in Brain Regions Responsible for Reward Processing

A significant increase in FC was found between the insula_L and pallidum_L. The insula functions as a central hub for inner perceptual awareness, processing bodily signals and satisfaction requirements, while modulating drugs that elicit cue‐driven addictions and cravings in certain circumstances, such as smoking and opiate consumption [58, 59, 60]. The pallidum receives efferent connections from the ventral striatum, especially from the nucleus accumbens, and sends a signal to the cortex via relay through the thalamus [61]. The pallidum is known to be associated mainly with motor functions, but its role in reward processing has also been widely discussed [62]. This could be attributed to the fact that short‐term abstinence elicited an acute compulsion to engage in gaming among individuals with IGD, resulting in increased activation of brain regions associated with executive control functions to manage cravings, alongside an escalation in cravings that increased activity in brain regions linked to reward functions.

### 4.3 Enhanced Coupling in Related Brain Regions Between Executive Control and Reward Function

In this study, we found a significant increase in FC values in the SFG_R and thalamus_R in the IGD group compared with those in the RGU group. The SFG is believed to play a role in the top‐down cognitive regulation of behaviours, encompassing decision‐making, selective attention, inhibitory control, desire and motor control [63, 64, 65]. The thalamus participates in reward processing by relaying information to the dopamine regulatory system to coordinate activity [66, 67]. These findings might be explained by the fact that, in contrast to those with substance addiction disorders such as alcohol and tobacco addiction, patients with IGD do not intentionally consume external substances [68]. This increased connectivity could be linked to the abstinence context, potentially reflecting enhanced top‐down regulation of craving or the activation of game‐related planning. Whether it represents an adaptive or maladaptive change remains to be determined.

### 4.4 Compared With Females, Males in the IGD Group Have Greater Functional Connections Between Executive Control and Reward

Compared with IGD females, IGD males presented significantly greater FC values in the SFG, MFG, OFC, PHG, precentral gyrus and ACC cortex. The MFG, specifically the DLPFC, is believed to play a role in self‐regulation, impulse control and reward systems and is assumed to modulate goal‐directed and habitual behaviours [69]. The MFG is believed to play a significant role in coordinating several systems and may also be linked to improved sensory–motor coordination [70, 71]. Abnormal functioning and structure of the OFC have been proposed as markers for individuals with IGD, while it is posited that the OFC integrates experiential history with current events to generate and sustain expectations of potential rewards associated with reinforcement [43, 72]. The PHG is chiefly accountable for reward memory and is believed to play a role in learning from prior experiences [73, 74, 75]. It has been proposed that functional irregularities in the PHG may be associated with the establishment of addiction memories in individuals with IGD [76]. The precentral gyrus may be associated with exposure to prospective loss when individuals must exert greater effort to evaluate a probable loss, leading to increased activation in the precentral gyrus [77, 78]. The ACC, as a principal core region and a focal point of selective attention [79], participates in various cognitive functions, including those related to cognitive regulation, conflict monitoring and error processing [80, 81]. The greater FC in males may reflect a distinct neural engagement during abstinence, not necessarily dysfunction. The aforementioned results indicate sex differences in the functional organization of control and reward networks [82], in contrast to findings from a prior article on task‐state fMRI [83], which demonstrated that the activation of brain regions associated with executive control functions (including the SFG and ACC cortex) was diminished in IGD males compared with IGD females under game cues. This discrepancy may arise from the variance between the game cue state and the short‐term abstinence state, resulting in opposing outcomes. This finding also corroborates the notion presented in prior studies that the neural activation state of the brain during short‐term abstinence may differ from that in the typical state [84].

The FC values of IGD males in the amygdala, precuneus, thalamus, caudate and dorsal striatum were similarly greater than those of IGD females. Altered functional connectivity in the amygdala affects self‐regulation, which is essential for managing cravings and significantly contributes to the onset and persistence of addictions [85], as well as influencing reward stimuli and associated emotional reactions [86]. The precuneus is integral to visual processing, attention and stimulus tracking [87], and it is involved in reward processing by facilitating the retrieval and integration of cues linked to past experiences and current addictions, transmitting this information to the prefrontal cortex [88, 89, 90]. The thalamus participates in reward processing by relaying information to the dopamine regulatory system to coordinate activity [66, 67]. The dorsal striatum, encompassing the nucleus accumbens and the caudate, is a crucial brain region for dopamine transmission and is utilized to reinforce reward expectations [91]. It functions as a part of the reward circuitry and may enhance motivation to seek rewards [56, 92]. Research indicates that increased activity in the caudate nucleus is correlated with heightened motivation to engage in play [93]. These findings align with prior hypotheses suggesting that males may exhibit more sensitivity to rewards than females [75]. A study on smoking addiction indicated that tobacco use disorder males exhibited greater activity in the hippocampus and amygdala in response to smoking cues than did tobacco use disorder females [94].

### 4.5 Intergroup Differences Exist Between Males and Females in Internal Control, Internal Reward and Internal Control and Reward

ANOVA and post hoc analyses revealed that IGD males presented increased functional connectivity in brain regions, including the IFG, MFG, SFG, precuneus, precentral gyrus, ACC, insula and PHG, relative to RGU males, whereas IGD females presented diminished functional connectivity in regions, such as the IFG, SFG, MFG, precuneus, precentral gyrus, insula and PHG, compared with RGU females. The current findings reveal notable sex disparities in executive control functions and reward processing during short‐term abstinence. A prior resting‐state fMRI investigation revealed that IGD males exhibited diminished ReHo activation in the PCC relative to RGU males, with no discernible difference between IGD females and RGU females [95]. In contrast, the notable intergroup disparity observed in the current findings may be attributed to the utilization of a greater number of resting‐state indicators in this study, thereby enhancing the precision of the results. In a task‐state fMRI investigation of forced abstinence, FC values were significantly elevated in both male and female patients with IGD compared with RGU, with a pronounced difference in FC observed among female subjects [19]. In a separate investigation on substance addiction utilizing task‐state fMRI, individuals with cocaine use disorder typically exhibited worse functional connectivity than healthy control volunteers [96]. Variations in outcomes may result from the differential effects of short‐term abstinence on sex, necessitating further investigation and discourse.

## 5 Limitations

Numerous constraints exist. The quantity of selected ROIs was constrained. While ROIs were chosen on the basis of prior research and their possible involvement in desire, executive control and reward processing, alternative ROIs or methodologies may produce divergent outcomes. Second, the conceptualization and duration of short‐term abstinence from the IGD remain contentious, and we anticipate increasingly precise and refined criteria. Additionally, the duration of gaming abstinence prior to the laboratory session was not controlled or recorded, which may have led to variability in baseline states before the standardized 90‐min washout period. Moreover, the induction of craving as a core withdrawal symptom during this 90‐min abstinence period was not directly validated with multi‐timepoint behavioural or physiological measures in the current study. Although the sample size of this trial was sufficient and sex‐matched, our control group was derived from RGUs instead of individuals with no gaming experience, which constrained the generalizability of the findings. Future studies may involve a comparative analysis of the three groups (IGD/RGU/HC) to identify differences.

## 6 Conclusions

The results of this research indicate that, following a short period of abstinence from internet gaming, individuals with IGD exhibit abnormal alterations in brain function within regions associated with reward and executive control, indicating an excessive desire for gaming and reward acquisition, coupled with a diminished capacity to regulate this intense craving due to inadequate executive control capabilities. In IGD males, organic alterations were observed in brain regions associated with the reward system and executive control functions, in contrast to IGD females.

## Author Contributions

**Shaoyu Cui:** writing – original draft, writing – review and editing, visualization, validation, data curation, formal analysis. **Xuefeng Xu:** conceptualization, methodology, software, data curation. **Guangheng Dong:** writing – review and editing, project administration, supervision, funding acquisition.

## Funding

The current research was supported by the Innovation Team Program in Philosophy and Social Science of Yunnan Province (research on psychological adaptation and development of China's ethnic minority students in border areas) and the Technology Talent and Platform Plan of Yunnan Province Science and Technology Department (202405AC350075).

## Ethics Statement

The study was approved by the Institutional Ethics Committee of Yunnan Normal University. All participants provided written informed consent prior to participation. The study was conducted in accordance with the 1964 Declaration of Helsinki and its later amendments.

## Consent

All authors have read and approved the manuscript for publication and consent to its submission. No individual data requiring separate consent for publication are included.

## Conflicts of Interest

The authors declare no conflicts of interest.

## Supporting information


**Table S1:** The results of the interaction between sex and group analysis of ANOVA.
**Table S2:** The results of simple effects analysis (differences between males and females in IGD).
**Table S3:** The results of simple effects analysis (differences between IGD and RGU in males).
**Table S4:** The results of simple effects analysis (differences between IGD and RGU in females).

## Data Availability

The data that support the findings of this study are available from the corresponding author upon reasonable request.
