# Supplementary material for: Sex Difference in Brain Responses During Short Abstinence in People With Internet Gaming Disorder
Source: Addict Biol. 2026 Mar 25;31(4):e70145. doi: 10.1111/adb.70145 (PMC13140647; doi:10.1111/adb.70145)
Supplement: Supplementary file 1 — Table S1: The results of the interaction between sex and group analysis of ANOVA. Table S2: The results of simple effects analysis (differences between males and females in IGD). Table S3: The results of simple effects analysis (differences between IGD and RGU in males). Table S4: The results of simple effects analysis (differences between IGD and RGU in females). [file ADB-31-e70145-s001.docx]

SUPPLEMENTARY INFORMATION

## Sex difference in brain responses during short abstinence in people with internet gaming disorder

Table S1. The results of the interaction between sex and group analysis of ANOVA

| ROI | Region | *F_1，116_* | *P* | *partial η²* |
| --- | --- | --- | --- | --- |
| IFG_L | MFG_L | 6.118 | .015^*^ | 0.05 |
| IFG_L | PHG_R | 8.05 | .005^**^ | 0.065 |
| IFG_L | SN_pc_L | 14.356 | .000^***^ | 0.11 |
| IFG_L | MFG_R | 6.812 | .010^*^ | 0.055 |
| IFG_L | SFG_L | 8.437 | .004^**^ | 0.068 |
| IFG_L | PCUN_L | 15.72 | .000^***^ | 0.119 |
| Insula_L | SFG_R | 6.753 | .011^*^ | 0.055 |
| Insula_L | PHG_L | 10.7 | .001^**^ | 0.084 |
| MFG_L | PHG_L | 11.594 | .001^**^ | 0.091 |
| MFG_L | IFG_L | 16.08 | .000^***^ | 0.122 |
| SFG_R | PHG_R | 6.837 | .010^*^ | 0.056 |
| SFG_R | Insula_R | 6.54 | .012^*^ | 0.053 |
| SFG_R | PreCG_R | 11.198 | .001^**^ | 0.088 |
| SFG_R | MFG_L | 6.004 | .016^*^ | 0.049 |
| SFG_R | ACC_L | 6.471 | .012^*^ | 0.053 |
| SFG_R | PHG_L | 14.621 | .000^***^ | 0.112 |

Abbreviation: ROI: Region of interest; IFG: Inferior frontal gyrus; INS: Insula; MFG: Middle frontal gyrus; SFG: Superior frontal gyrus; SN_pc: Substantia nigra, pars compacta; PCUN: Precuneus; PHG: Parahippocampal gyrus; ACC: Anterior cingulate; PreCG: Precentral gyrus. * representative *p*<0.05, ** representative *p*<0.01, *** representative *p*<0.001.

Table S2. The results of simple effects analysis (differences between males and females in IGD)

| ROI | Region | *F_1，116_* | *P* | *Cohen d* |
| --- | --- | --- | --- | --- |
| IFG_L | MFG_L | 6.506 | .015^*^ | 0.658 |
| IFG_L | PHG_R | 10.846 | .003^**^ | 0.851 |
| IFG_L | SN_pc_L | 20.622 | .000^***^ | 1.173 |
| IFG_L | MFG_R | 12.406 | .003^**^ | 0.91 |
| IFG_L | SFG_L | 25.425 | .000^***^ | 1.302 |
| IFG_L | PCUN_L | 25.804 | .000^***^ | 1.311 |
| Insula_L | SFG_R | 11.881 | .003^**^ | 0.89 |
| Insula_L | PHG_L | 20.777 | .000^***^ | 1.178 |
| MFG_L | PHG_L | 17.156 | .000^***^ | 1.069 |
| MFG_L | IFG_L | 13.782 | .000^***^ | 0.958 |
| SFG_R | PHG_R | 13.843 | .000^***^ | 0.961 |
| SFG_R | Insula_R | 19.125 | .000^***^ | 1.13 |
| SFG_R | Precentral_R | 10.541 | .003^**^ | 0.838 |
| SFG_R | MFG_L | 13.185 | .000^***^ | 0.937 |
| SFG_R | ACC_L | 2.973 | .087^*^ | 0.445 |
| SFG_R | PHG_L | 15.08 | .000^***^ | 1.003 |

Abbreviation: ROI: Region of interest; IFG: Inferior frontal gyrus; INS: Insula; MFG: Middle frontal gyrus; SFG: Superior frontal gyrus; SN_pc: Substantia nigra, pars compacta; PCUN: Precuneus; PHG: Parahippocampal gyrus; ACC: Anterior cingulate. * representative *p*<0.05, ** representative *p*<0.01, *** representative *p*<0.001.

Table S3. The results of simple effects analysis (differences between IGD and RGU in males)

| ROI | Region | *F_1，116_* | *P* | *Cohen d* |
| --- | --- | --- | --- | --- |
| IFG_L | MFG_L | 3.937 | .050^*^ | 0.512 |
| IFG_L | PHG_R | 7.598 | .007^**^ | 0.712 |
| IFG_L | SN_pc_L | 6.59 | .012^*^ | 0.663 |
| IFG_L | MFG_R | 4.017 | .047^*^ | 0.517 |
| IFG_L | SFG_L | 5.628 | .019^*^ | 0.612 |
| IFG_L | PCUN_L | 9.041 | .003^**^ | 0.776 |
| Insula_L | SFG_R | 7.18 | .011^*^ | 0.692 |
| Insula_L | PHG_L | 6.383 | .017^*^ | 0.653 |
| MFG_L | PHG_L | 4.143 | .044^*^ | 0.526 |
| MFG_L | IFG_L | 17.129 | .000^***^ | 1.069 |
| SFG_R | MFG_L | 5.082 | .026^*^ | 0.582 |
| SFG_R | ACC_L | 4.748 | .031^*^ | 0.562 |
| SFG_R | PHG_L | 4.933 | .028^*^ | 0.574 |

Abbreviation: ROI: Region of interest; IFG: Inferior frontal gyrus; INS: Insula; MFG: Middle frontal gyrus; SFG: Superior frontal gyrus; SN_pc: Substantia nigra, pars compacta; PCUN: Precuneus; PHG: Parahippocampal gyrus; ACC: Anterior cingulate. * representative *p*<0.05, ** representative *p*<0.01, *** representative *p*<0.001.

Table S4. The results of simple effects analysis (differences between IGD and RGU in females)

| ROI | Region | *F_1，116_* | *P* | *Cohen d* |
| --- | --- | --- | --- | --- |
| IFG_L | SN_pc_L | 7.791 | .009^**^ | -0.721 |
| IFG_L | PCUN_L | 6.762 | .011^*^ | -0.671 |
| Insula_L | PHG_L | 4.408 | .038^*^ | -0.542 |
| MFG_L | PHG_L | 7.727 | .008^**^ | -0.717 |
| SFG_R | PHG_R | 7.837 | .006^**^ | -0.723 |
| SFG_R | Insula_R | 4.144 | .044^*^ | -0.526 |
| SFG_R | Precentral_R | 9.133 | .003^**^ | -0.78 |
| SFG_R | PHG_L | 10.155 | .003^**^ | -0.822 |

Abbreviation: ROI: Region of interest; IFG: Inferior frontal gyrus; INS: Insula; MFG: Middle frontal gyrus; SFG: Superior frontal gyrus; SN_pc: Substantia nigra, pars compacta; PCUN: Precuneus; PHG: Parahippocampal gyrus; ACC: Anterior cingulate. * representative *p*<0.05, ** representative *p*<0.01, *** representative *p*<0.001.
